# PB.7. Large-volume biopsy for B3 lesions without atypia: is once enough?

**DOI:** 10.1186/bcr3715

**Published:** 2014-11-03

**Authors:** N Forester

**Affiliations:** 1 Breast Screening and Assessment Unit, Newcastle, UK

## Introduction

B3 lesion incidence has increased with digital mammography and screening of younger women. In January 2012 we changed management of B3 lesions, following pathways published by Rajan and colleagues. This study reviews the outcome of vacuum-assisted biopsy (VAB) in all patients with a B3 diagnosis, comparing initial core and subsequent VAB for concordance.

## Methods

A single-centre, prospective study of all B3 lesions from January 2012 to June 2014 diagnosed by 14G (US) or 7 to 10G (stereotactic) biopsy with subsequent 7 to 8G VAB.

## Results

Over 30 months, 258 B3 lesions were identified in 255 women. Second-line VAB was performed for 192 lesions (66 no further intervention/excision biopsy at pathologist request). In total, 79/192 were lesions without atypia (fibroepithelial/papillary/RS/CSL) from which three were upgraded by VAB (two B5a, one B4). All of the upgraded B3 lesions without atypia had an initial 14G core. In total, 113/192 were lesions with atypia (ADH/AIDP/FEA/LISN/LCIS/papillary/RS/CSL) from which 21 were upgraded by VAB (18%). VAB and initial histology were concordant in 87% of lesions.

## Conclusion

VAB in B3 lesions with atypia has a significant and important upgrade rate to malignancy. However, in the absence of atypia the upgrade rate is very low (<4%). In this group, there was no discordant pathology between an initial 10G stereotactic core and subsequent 7 to 8G large-volume biopsy. To reduce unnecessary intervention and overdiagnosis, we would like to propose that one 10G biopsy is sufficient for B3 lesions without atypia, but that lesions identified on 14G core and those with atypia should continue to have a second-line VAB performed.

